# Concomitant magnetic-field compensation for 2D spiral-ring turbo spin-echo imaging at 0.55T and 1.5T

**DOI:** 10.1002/mrm.29663

**Published:** 2023-04-10

**Authors:** Zhixing Wang, Rajiv Ramasawmy, Xue Feng, Adrienne E. Campbell-Washburn, John P. Mugler, Craig H. Meyer

**Affiliations:** 1 Department of Biomedical Engineering, University of Virginia, Charlottesville, Virginia, USA; 2 Cardiovascular Branch, Division of Intramural Research, National Heart, Lung, and Blood Institute, National Institutes of Health, Bethesda, Maryland, USA; 3 Department of Radiology & Medical Imaging, University of Virginia, Charlottesville, Virginia, USA

**Keywords:** concomitant gradient field, fast imaging, spiral imaging, turbo spin-echo imaging

## Abstract

**Purpose:**

To develop 2D turbo spin-echo (TSE) imaging using annular spiral rings (abbreviated “SPRING-RIO TSE”) with compensation of concomitant gradient fields and $B_{0}$ inhomogeneity at both 0.55T and 1.5T for fast T_2_-weighted imaging.

**Methods:**

Strategies of gradient waveform modifications were implemented in SPRING-RIO TSE for compensation of self-squared concomitant gradient terms at the TE and across echo spacings, along with reconstruction-based corrections to simultaneously compensate for the residual concomitant gradient and $B_{0}$ field induced phase accruals along the readout. The signal pathway disturbance caused by time-varying and spatially dependent concomitant fields was simulated, and echo-to-echo phase variations before and after sequence-based compensation were compared. Images from SPRING-RIO TSE with no compensation, with compensation, and Cartesian TSE were also compared via phantom and in vivo acquisitions.

**Results:**

Simulation showed how concomitant fields affected the signal evolution with no compensation, and both simulation and phantom studies demonstrated the performance of the proposed sequence modifications, as well as the readout off-resonance corrections. Volunteer data showed that after full correction, the SPRING-RIO TSE sequence achieved high image quality with improved SNR efficiency (15%–20% increase), and reduced RF SAR (~50% reduction), compared to the standard Cartesian TSE, presenting potential benefits, especially in regaining SNR at low-field (0.55T).

**Conclusion:**

Implementation of SPRING-RIO TSE with concomitant field compensation was tested at 0.55T and 1.5T. The compensation principles can be extended to correct for other trajectory types that are time-varying along the echo train and temporally asymmetric in TSE-based imaging.

## 1 | INTRODUCTION

Concomitant fields, also known as Maxwell fields, created simultaneously with the spatially linear gradient fields, are a potential source of artifacts in MR imaging.^1^ The presence of concomitant fields causes undesired phase accumulation that may contaminate the measured k-space data. If not properly accounted for in pulse sequence design and/or image reconstruction, this unwanted phase accrual may lead to severe signal dropouts, image blurring, or ghosting artifacts in a variety of imaging methods, such as turbo spin-echo (TSE) imaging,^2^ spiral imaging,^3^ and EPI.^4,5^

T_2_-weighted TSE imaging is a workhorse pulse sequence for clinical practice because of its fast scan speed and high sensitivity to many pathologies.^6,7^ However, phase errors among echoes induced by system imperfections (e.g., eddy currents, concomitant fields) can produce artifacts. Since the gradients used for imaging inevitably generate concomitant fields, which scale quadratically with gradient amplitude, compensating for them becomes increasingly important for TSE when using readouts with high amplitudes (>20 mT/m). There has recently been renewed interest in MRI at lower magnetic field strengths (<1T),^8,9^ where these effects are increased because concomitant fields scale inversely with field strength, and thus the phase errors induced by concomitant gradients increase. Concomitant gradient effects in Cartesian TSE have been well described, and several strategies have been developed, for example, as described by Zhou et al.,^2^ to eliminate or minimize the associated image degradation.

Spiral-based TSE imaging has been shown to have advantages over conventional Cartesian TSE at 3T, in terms of SNR efficiency, improved image contrast, and reduced specific absorption rate (SAR).^10–13^ Imaging with prolonged readouts, such as spiral acquisitions at low fields, have recently shown benefits in regaining SNR using a high-performance MR scanner at 0.55T,^8,14–16^ because $B_{0}$ off-resonance effects decrease as field strength decreases. Therefore, a potentially important application area for spiral TSE is low field imaging. However, spiral TSE imaging presents challenges for compensating concomitant gradient effects, since the spiral readouts vary along the echo train as opposed to Cartesian TSE, where the same readout waveform is used for every echo. Hence, concomitant field phase errors induced by differences in spiral readouts along the echo train may disturb the TSE signal pathways and violate the Carr-Purcell-Meiboom-Gill (CPMG) condition,^17^ resulting in severe signal loss and image blurring which cannot be fully corrected in image reconstruction. Researchers have investigated concomitant gradient compensation for interleaved, time-symmetric spiral-in-out TSE imaging,^12,18^ and recently Mugler et al.^19^ redesigned the pulse sequence to achieve compensation of self-squared Maxwell field terms by modifying gradient waveforms along the entire echo train for 2D T_2_-weighted interleaved, rotated spiral-arm TSE imaging with several trajectory types (spiral-out, –in, or -in-out). Promising results showed that this approach provided substantial improvement in image quality at 0.55T by reducing or eliminating degradation associated with self-squared concomitant gradient effects.

Our previous work proposed an alternative approach to 2D TSE imaging using annular spiral rings with a retraced in/out trajectory, dubbed “SPRING-RIO TSE”, for fast T_2_-weighted brain imaging at 3T.^10^ Compared to the interleaved, rotated spiral-arm segmentation which requires a double-encoding strategy,^12^ annular spiral-ring segmentation inserts several annular segments into TSE echoes, with the benefit of reduced T_2_-decay artifacts by converting the T_2_-dependent signal modulation into a k-space apodizing filter.^10,20,21^ Since spiral-ring waveforms for each echo vary along the echo train and are temporally asymmetric, concomitant field effects on images from SPRING-RIO TSE at relatively low-field strength become nonnegligible and must be corrected.

This study proposes a general method that incorporates both pulse sequence design and image reconstruction methods to compensate for concomitant field effects in SPRING-RIO TSE.^22^ First, we introduce strategies for gradient waveform modifications to compensate for the self-squared Maxwell terms at the TE and between echo spacings. Second, we describe image reconstruction methods to correct for residual concomitant fields and $B_{0}$ inhomogeneity induced phase accruals along the readout. Finally, we validate the feasibility of the proposed method and compare its performance to that of SPRING-RIO TSE with no concomitant field compensation and Cartesian TSE in phantom and in vivo scans at both 0.55T and 1.5T.

## 2 | METHODS

### 2.1 | Concomitant field corrections

The mathematical description of Maxwell fields $B_{c}$ can be estimated based on the following equation,^1^ omitting the third and higher order terms,

(1) $$B_{c}\left(g_{x},g_{y},g_{z},x,y,z\right)=\left(\frac{g_{z}^{2}}{8B_{0}}\right)\left(x^{2}\;+\;y^{2}\right)+\left(\frac{g_{x}^{2}\;+\;g_{y}^{2}}{2B_{0}}\right)z^{2}\;\;-\left(\frac{g_{x}g_{z}}{2B_{0}}\right)xz-\left(\frac{g_{y}g_{z}}{2B_{0}}\right)yz$$

where x,y, and z are the laboratory coordinates, and $B_{0},\;g_{x},\;g_{y}$, and $g_{z}$ are the static field and the readout gradients in the laboratory system, respectively. The first two terms are self-squared terms, and the last two are quadratic cross-terms. Ignoring relaxation and considering the presence of both $B_{0}$ inhomogeneities and concomitant fields, for $t\in \left[-\frac{\tau }{2},\frac{\tau }{2}\right]$, the received MR signal s(t) from an object is given by:

(2) $$s\left(t\right)=\int \;m\left(r\right)e^{-i2\pi k\left(t\right)\cdot r}e^{-i\left(\Delta \omega \left(r\right)t+\phi_{c}\left(\Delta \omega_{c}\left(r\right),t\right)\right)}dr$$

where k(t) is the k-space trajectory, and m(r) is the complex-valued magnetization. τ is the readout time, Δω is the frequency offset of $B_{0}$ field inhomogeneities, and $\phi_{c}\left(\Delta \omega_{c}(r),t\right)$ represents the phase accruals due to concomitant fields $B_{c}$.

To produce high quality spiral-ring TSE images at 0.55T and 1.5T, the concomitant field induced phase error must be eliminated or mitigated to a negligible level. In this work, several strategies are presented to substantially reduce the phase errors in SPRING-RIO TSE via both pulse sequence modifications and the image reconstruction process.

#### 2.1.1 | Sequence-based corrections

The goal of gradient waveform modifications is to eliminate the phase shift from self-squared terms at the k-space center, and to reduce the difference in phase shifts among echoes, targeting a constant phase shift at the end of every echo. For any arbitrary gradient $g^{\prime }(t)$, its waveform can be decomposed into three orthogonal gradient components $\left\{g_{x}^{\prime }(t),g_{y}^{\prime }(t),g_{z}^{\prime }(t)\right\}$, and the concomitant field integral $M=\left\{M_{x},M_{y},M_{z}\right\}$ of each component from self-squared terms can be calculated as:

(3) $$M=\left\{\int \;{\left(g_{x}^{\prime }\left(t^{\prime }\right)\right)}^{2}dt^{\prime },\;\int \;{\left(g_{y}^{\prime }\left(t^{\prime }\right)\right)}^{2}dt^{\prime },\int \;{\left(\frac{g_{z}^{\prime }\left(t^{\prime }\right)}{2}\right)}^{2}dt^{\prime }\right\}$$

For a single spatial location $r_{0}$, the phase error from the self-squared terms induced by the concomitant field can be determined by its concomitant field integral M.

**FIGURE 1:**
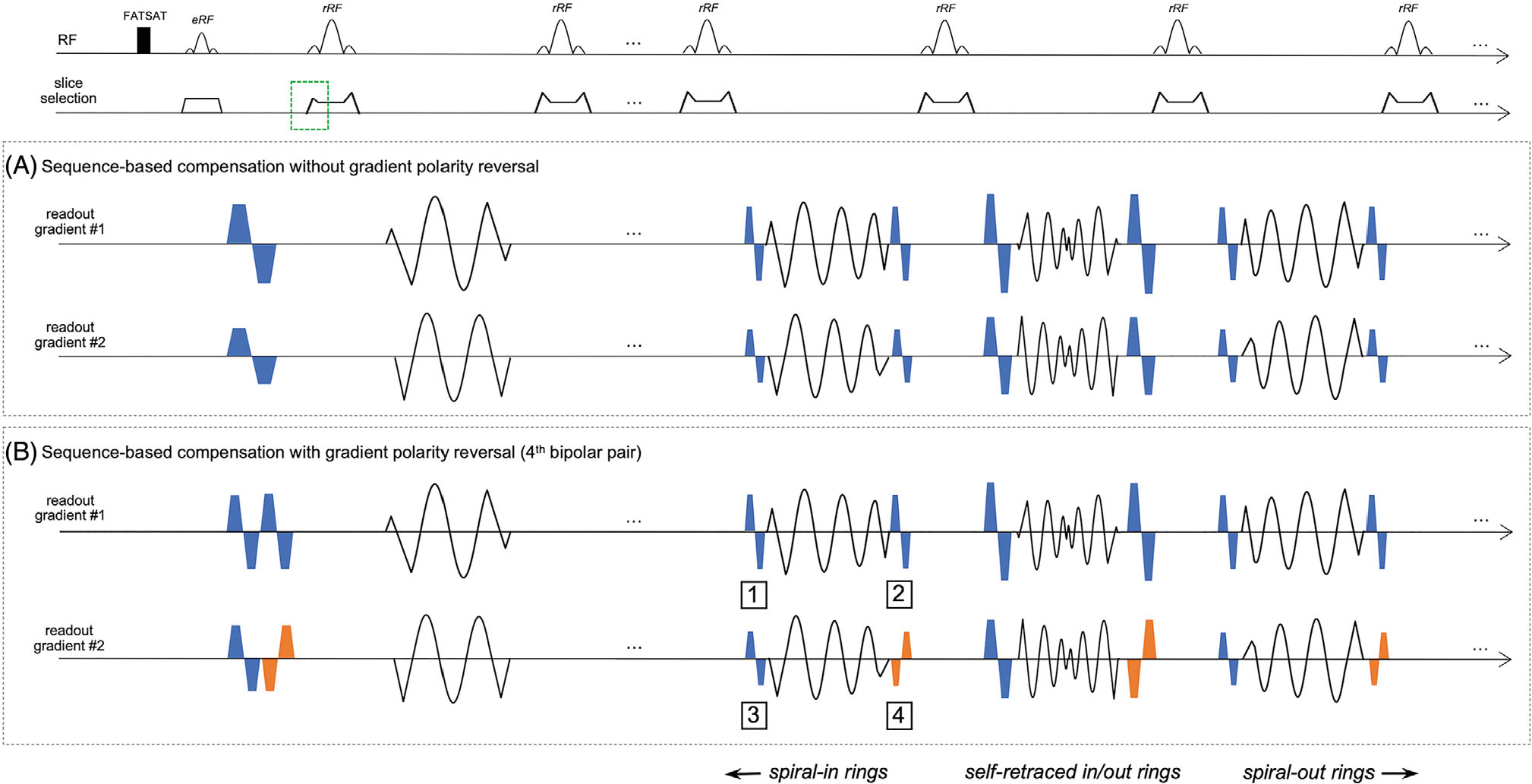
Pulse sequence timing diagrams including fat saturation, TSE data acquisition using annular spiral rings, a reshaped gradient waveform for slice-selection (green dashed box), and additional bipolar gradients (blue boxes) placed at each readout gradient axis 1 and 2 for concomitant field compensation along the echo train. For each shot, the data were collected by spiral-in rings, a self-retraced spiral in-out ring, and spiral-out rings, sequentially, with the number of spiral-in rings equivalent to that of spiral-out rings. Inner rings require larger bipolar gradients than outer rings for maintaining the constant concomitant self-squared terms at the end of each echo spacing. (A) Sequence-based compensation without bipolar-gradient polarity reversal. (B) Sequence-based compensation with bipolar-gradient polarity reversal. Compared to A, the gradient polarity of one bipolar gradient pair in each echo spacing, the fourth pair for example (orange boxes), is set to be the opposite of the other pairs for self-balancing the concomitant quadratic cross-terms induced by these four added bipolar gradients. The first two bipolar pairs placed between the excitation RF pulse and the first refocusing RF pulse shown in A are split into four pairs, followed by the gradient polarity reversal of the fourth pair. A total of 5 ms additional time is added for an increased echo spacing to both sequences shown in A and B.

In SPRING-RIO TSE, the data were collected by self-retraced spiral in-out rings for the center of k-space, spiral-out rings at the end of the echo train paired with time-reversed, spiral-in rings with opposite gradient polarity at the beginning of the echo train for the outer portion of k-space. Therefore, the implementation of sequence shown in Figure 1 was accomplished as follows:

1. The gradient waveform reshaping strategy, as described in Ref. [2], was used to simultaneously nullify both the linear and the quadratic phase induced by encoding gradients and their concomitant gradients, respectively. In this work, the left crusher gradient of the first refocusing RF pulse (green dashed box) was redesigned to correct for echo-to-echo phase variations from self-squared terms produced by the slice selection gradients/crushers.
2. For each excitation (shot), the maximum concomitant field integral $M_{max}$ from self-squared terms for each readout gradient axis was determined from the spiral-ring with the highest gradient amplitude (see Figure S1).
3. Bipolar gradient pairs were added at both the beginning and end of each remaining echo spacing (i.e., two pairs for each axis) to increase the concomitant field integrals for each gradient axis. The gradient amplitudes and durations of the added bipolar pairs for each echo were determined by subtraction of the concomitant field integral $M_{j}$ for the current $j^{\text{th}}$ spiral-ring from $M_{max}$, while those of the bipolar pairs placed at the interval between the excitation RF pulse and the first refocusing RF pulse were determined by $\frac{M_{max}}{2}$.
4. The gradient polarity of one of the four bipolar pairs in each echo spacing was set to be the opposite of the others (Figure 1B) for self-balancing quadratic cross-terms induced by added bipolar pairs themselves. The benefits of this strategy compared to that without the gradient polarity reversal (Figure 1A) will be discussed in the following sections.
5. Additional time was added to each echo spacing as needed to achieve compensation, and the final concomitant integral at the end of each echo spacing was designed to be a constant value of $\frac{M_{max}}{2}$. In this work, 5 ms of additional time (2.5 ms before and after each readout) was added for bipolar gradient pairs. This additional time was also added to SPRING-RIO TSE with no compensation for comparison to that with sequence-based concomitant field corrections.

#### 2.1.2 | Reconstruction-based corrections

The goal of the image reconstruction method is to further reduce the residual phase errors from concomitant gradients and $B_{0}$ off-resonance effects accrued during the readout. As reported in Ref. [3], for spiral scanning, the concomitant gradient phase accruals along the acquisition window were approximated from the lowest order Maxwell gradients for arbitrary scan plane orientation as follows:

(4) $$\phi_{c}\left(\Delta \omega_{c}(r),t\right)=\Delta \omega_{c}(r)t_{c}$$

with

(5) $$\Delta \omega_{c}(r)=\gamma \frac{g_{m}^{2}}{4B_{0}}\left(F_{1}X^{2}+F_{2}Y^{2}+F_{3}Z^{2}\right.\;\;\left.+F_{4}XZ+F_{5}YZ+F_{6}XY\right)$$

and a scaled concomitant field time parameter $t_{c}(t)$ given by:

(6) $$t_{c}\left(t\right)=\frac{1}{g_{m}^{2}}\int_{0}^{t}\;g_{0}^{2}\left(t^{\prime }\right)dt^{\prime }$$

where $g_{0}$ is the gradient envelope and $g_{m}$ is the maximal readout gradient amplitude used in all spiral rings. X,Y,Z are the logical coordinates which define the readout 1, readout 2, and slice coordinates, respectively. $F_{i}$ are constants calculated from the rotation matrix, which are given in the appendix in Ref. [3]. In TSE imaging, however, each refocusing RF pulse alternates the sign of the accumulated Maxwell phase, resulting in a negative phase from the self-squared terms at the beginning and a positive phase at the end of each echo spacing (ESP). Hence, we modified the time parameter $t_{c_{j}}(t)$ specifically for SPRING-RIO TSE, as:

(7) $$t_{c_{j}}\left(t\right)=\frac{1}{g_{m}^{2}}\left(\int_{0}^{t}\;\;g_{j}^{2}\left(t^{\prime }\right)dt^{\prime }-\frac{M_{j}}{2}\right)$$

where $g_{j}$ is the gradient envelope and $M_{j}$ is the concomitant field integral of the $j^{\text{th}}$ ring trajectory. The derivation of Eq. 7 can be found in the Appendix A.

Previously, a semiautomatic deblurring method with a maximized energy objective^10,23^ was applied to SPRING-RIO TSE for $B_{0}$ field inhomogeneity correction. The term “semiautomatic” refers to the method where an automatic method is used to search for a high-resolution field map using offset frequency constraints calculated from an acquired low-resolution map. In this work, we chose the fast conjugate phase reconstruction method based on a Chebyshev approximation proposed by Chen et al.^24^ and extended it to correct for nonlinear off-resonance effects induced by both $B_{0}$ field inhomogeneities and concomitant gradients in SPRING-RIO TSE. To perform simultaneous semiautomatic $B_{0}$ off-resonance correction and concomitant gradient compensation, a series of images are reconstructed from the following equation:

(8) $$m\left(r;\Delta \omega_{i}\right)=\sum_{k=0}^{N-1}h_{k}\left(\Delta \tilde{\omega }(r)+\Delta \omega_{i},\Delta \omega_{c}(r),\tau \right)I_{k}(r)\;\;-\frac{1}{2}h_{0}I_{0}(r)\;i=1,2\ldots$$

where $\Delta \tilde{\omega }(r)$ is the $B_{0}$ off-resonance frequency constraint calculated from a low-resolution field map, and $\Delta \omega_{i}$ is constant frequency shift from $\Delta \tilde{\omega }(r)$. $h_{k}$ is the constant Chebyshev coefficient as a function of the local $B_{0}$ inhomogeneity, the concomitant field, and the readout length τ, the calculation of which is given in the appendix in Ref. [24]. $I_{k}(r)$ is the $k^{\text{th}}$ order Chebyshev demodulated base image, and for $t\in \left[-\frac{\tau }{2},\frac{\tau }{2}\right]$, it can be calculated as follows:

(9) $$I_{k}\left(r\right)=\int \;{\left(\frac{2t}{\tau }\right)}^{k}W\left(t\right)s\left(t\right)e^{i2\pi k\left(t\right)\cdot r}dt,$$

where W(t) is the density compensation function. The concomitant field effect is first corrected when reconstructing the demodulated image $m\left(r;\Delta \omega_{i}\right)$, followed by a semiautomatic deblurring method for $B_{0}$ field inhomogeneity correction, using a maximized energy objective function:

(10) $$\max_{\Delta \omega_{i}}\int p\left(r-r^{\prime }\right)\;m\left(r^{\prime };\Delta \omega_{i}\right)\;m{\left(r^{\prime };\Delta \omega_{i}\right)}^{*}dr^{\prime }$$

where $m{\left(r;\Delta \omega_{i}\right)}^{*}$ is the complex conjugate of $m\left(r;\Delta \omega_{i}\right)$, and p(r) is the convolution kernel chosen to be a circularly symmetric Gaussian window. The optimization of $\Delta \omega_{i}$ that best deblurs a local region of $m\left(r;\Delta \omega_{i}\right)$ is performed by searching for a correct demodulated frequency which maximizes its local integral of signal energy. A high-resolution field map will then be generated after Eq. 10, each pixel of which has its own estimated constant frequency shift.

The total number of base images required depends on the range of $B_{0}$ inhomogeneity and concomitant gradient field. Linear $B_{0}$ off-resonance correction based on an estimated spatially linear field map was incorporated to reduce the computational cost by narrowing the range of $B_{0}$ field inhomogeneity. For a given scan plane orientation with FOV and table shifts, linear concomitant gradient correction was also applied to reduce the frequency range of an off-center slice to that of a slice at isocenter.^24^

### 2.2 | Simulations

All simulations were implemented in MATLAB (R2020b software; MathWorks, Natick, MA). To illustrate the Maxwell field effects from self-squared terms for the SPRING-RIO TSE sequence, signal pathways along the echo train at several off-center table (z) locations and in the presence of $B_{1}$ inhomogeneity (resulting in different refocusing RF flip angles) were simulated by extended phase graph^25^ (EPG) method with no k-space weighting. Signal intensity loss $\left({SI}_{l}\right)$ at each echo was calculated based on the equation:

(11) $${SI}_{l}=\frac{{SI}_{c}\;-\;{SI}_{r}}{{SI}_{r}}$$

where ${SI}_{c}$ and ${SI}_{r}$ are the normalized signal curve of the current scenario and the standard $T_{2}$-decay curve as the reference, respectively. Specifically, axial planes with off-center table locations ranging from 0 to 60 mm with 20 mm increments, and with the refocusing RF flip angles ranging from 120° to 180° with 20° increments, were used for simulation. Other simulation parameters included $B_{0}=0.55T$, echo train length $(ETL)=9,\;spiral\;readout=16\;ms,\;{\;T}_{1}=800\;ms$, and $T_{2}=70\;ms$.

Ignoring $B_{0}$ inhomogeneity, the simulation of phase evolutions from self-squared terms during the acquisition window and along the echo train was performed for SPRING-RIO TSE with and without sequence-based corrections, for a $z_{c}=50\;mm$ off-center axial plane at 0.55T. First, the net Maxwell-field-induced phase $\Delta \phi_{c_{j}}$ at the $j^{\text{th}}$ ring trajectory with a gradient envelope $g_{j}$ during the readout was calculated based on:

(12) $$\Delta \phi_{c_{j}}\left(t\right)=\frac{\gamma z_{c}^{2}}{2B_{0}}\int_{0}^{t}\;g_{j}^{2}\left(t^{\prime }\right)dt^{\prime }.$$

Second, considering the 180° refocusing RF pulse which alternates the sign of the Maxwell field induced phase, the accrued phase $\phi_{c_{j}}$ for $j^{\text{th}}$ ring trajectory is given by:

(13) $$\phi_{c_{j}}(t)=\sum_{k=0}^{j-1}{\left(-1\right)}^{k+j}\Delta \phi_{c_{k}}(\tau )+\Delta \phi_{c_{j}}(t),$$

where $\Delta \phi_{c_{0}}(\tau )$ is the net phase accrual between the excitation and first refocusing pulses. Finally, the increased phase accruals induced by the added bipolar gradients were also calculated and added into $\phi_{c_{j}}$ for each echo spacing in SPRING-RIO TSE with sequence-based modifications. Further, to illustrate the quadratic cross-terms and the effect of bipolar-gradient polarity reversal on the SPRING-RIO TSE sequence, Maxwell phase evolutions during two individual (second and central) echo spacings were simulated at a specific sagittal location with and without bipolar-gradient polarity reversal.

### 2.3 | MRI experiments

#### 2.3.1 | Data acquisition

Experiments were performed on 1.5T (MAGNETOM Avanto) and prototype 0.55T with high-performance gradients (ramped-down MAGNETOM Aera) MR scanners (Siemens Healthcare, Erlangen, Germany) using a 12-channel (1.5T) or 16-channel (0.55T) receive head coil.

In phantom studies, sagittal data from a resolution phantom was acquired at 0.55T using SPRING-RIO TSE with no compensation, with sequence-based compensation (Figure 1A), and with sequence-based compensation including bipolar-gradient reversal (Figure 1B). At 1.5T, images from an axial plane at isocenter were acquired using SPRING-RIO TSE with no compensation and Cartesian TSE as a reference, while images at −10.6 cm off-center location were acquired via SPRING-RIO TSE with no compensation and with sequence-based compensation as shown in Figure 1B. Relevant spiral imaging parameters included FOV = 180 × 180 mm^2^, spatial resolution = 0.65 × 0.65 mm^2^, slice thickness = 4mm, refocusing RF flip angle = 180°, ETL = 8 (0.55T) or 9 (1.5T), and sampling duration = 21 ms (0.55T) or 12 ms (1.5T).

Eleven healthy volunteers (six at 0.55T and five at 1.5T) gave informed consent and were scanned using SPRING-RIO TSE sequences with and without sequence-based compensation, and a standard Cartesian TSE sequence to evaluate overall image quality. Table 1 lists parameters of pulse sequences used for human studies at 0.55T and 1.5T. Data were acquired consecutively at matched imaging planes using 14 slices with 4-mm thickness and 2-mm gap. A saturation pulse was used for fat suppression, and for reconstruction corrected spiral-ring trajectories were utilized based on a one-time model-based trajectory calibration.^10,26^ Axial, coronal, and sagittal slices of the brain were scanned, with an increased FOV (250 × 250 mm^2^) for non-axial orientations. For signal averaging, the data of each slice from SPRING-RIO TSE was acquired once at 1.5T (1-NSA, 0:33min) and six times at 0.55T (6-NSA, 2:24min).

#### 2.3.2 | Image reconstruction

All images were reconstructed offline in MATLAB. 2D NUFFT^27^ code was used for non-Cartesian image reconstruction. ESPIRiT^28^ was used for coil sensitivity map estimation. A low-resolution $B_{0}$ field map was generated from single-shot spirals at two TEs (ΔTE=1ms) acquired during two preparation scans before TSE acquisitions.^29^ To illustrate the performance of reconstruction-based compensation, both phantom and brain images using SPRING-RIO TSE with sequence-based compensation were reconstructed and compared with no compensation, with concomitant field correction only, and with simultaneous concomitant field and $B_{0}$ field inhomogeneity corrections.

A 3D table of Chebyshev coefficients $h_{k}$ was precalculated with $B_{0}$ field inhomogeneity $\left(\frac{\Delta \omega_{i}}{2\pi }\right)$ ranging from −150 to 150 Hz (1.5T) or −80 to 80 Hz (0.55T), and concomitant field off-resonance frequency $\left(\frac{\Delta \omega_{c}}{2\pi }\right)$ ranging from 0 to 250 Hz(1.5T) or 0 to 400 Hz(0.55T), both with a 1 Hz frequency increment and 15 base images. As described in Ref. [24], the range of frequency was sufficient after incorporating linear concomitant field and $B_{0}$ field inhomogeneity corrections, and this 3D table was used for data sets acquired with similar sequence parameters. The searching range of $B_{0}$ field offset frequency shifts from −60 to 60 Hz with a 10 Hz frequency increment was used for the semiautomatic deblurring.

#### 2.3.3 | Image quality analysis

Evaluation of SPRING-RIO TSE with full compensation and conventional Cartesian TSE was performed quantitatively on in vivo data. SNR with the pseudo-replica method^30^ was calculated, and the SNR efficiency map was then derived by multiplying the calculated SNR by the coefficient $1/(voxel\;size\;\times \sqrt{\text{scan}\;\text{time}})$, which equaled 1.17 at 0.55T and 2.38 at 1.5T for SPRING-RIO TSE, and 1.0 at 0.55T and 1.66 at 1.5T for Cartesian TSE. Regions of interest (ROIs) were drawn in white matter and gray matter on the SNR efficiency maps, and the averaged SNR was obtained for each subject at both 0.55T and 1.5T, with three slices per orientation and a total of nine slices per subject. Pairwise comparisons between these two imaging methods were performed using one-way analysis of variance (ANOVA) with the Tukey–Kramer post hoc test.

## 3 | RESULTS

### 3.1 | Simulations

**FIGURE 2:**
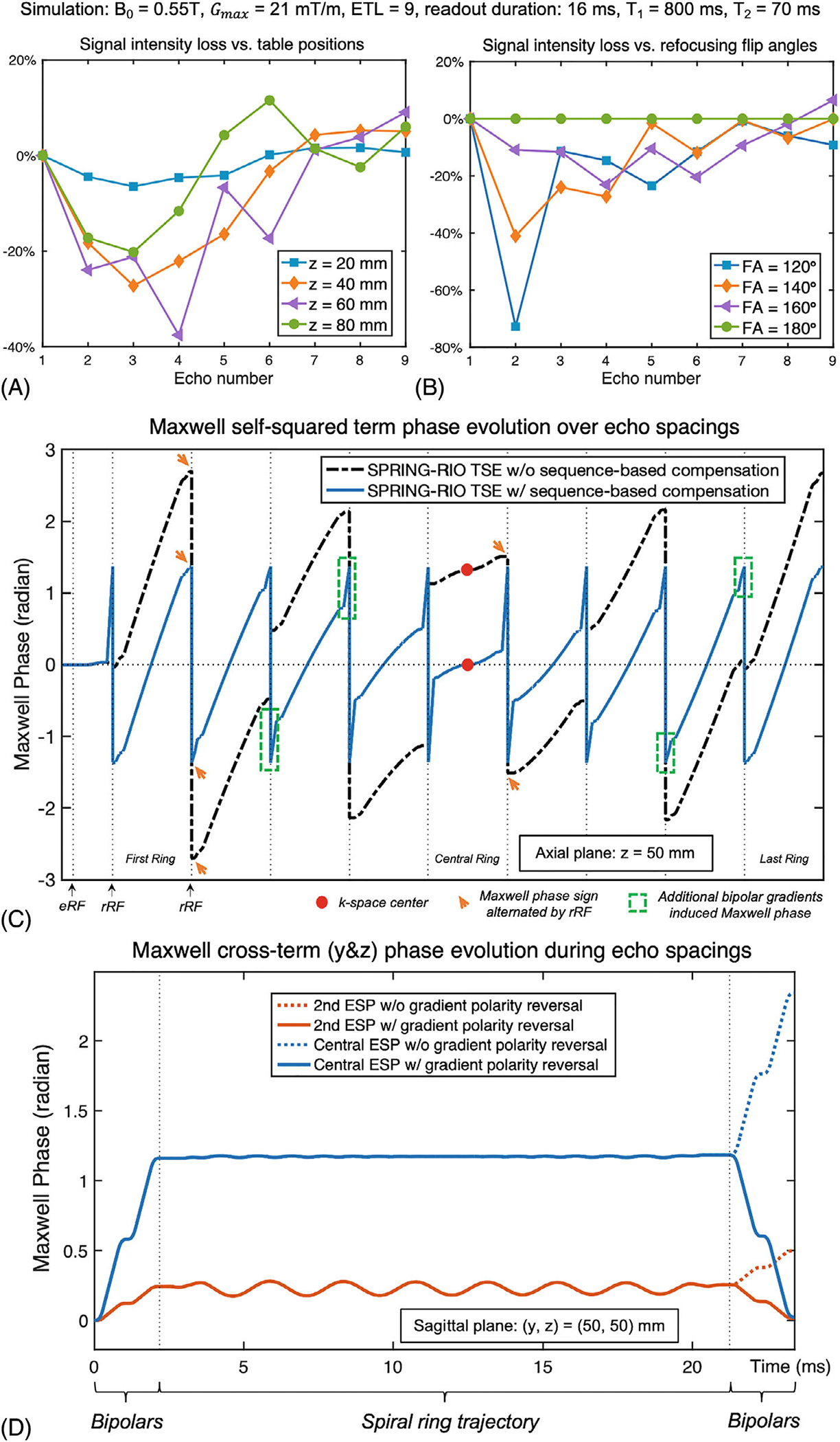
(A, B) Simulation results showing how Maxwell fields affect the signal pathway of the SPRING-RIO TSE sequence along the echo train, at several off-center axial (z) table locations (A) and with different refocusing RF flip angles (B). Note that each signal evolution was simulated without k-space weighting. (A) 150° refocusing RF flip angle was used for the simulation of A and a 40 mm off-center axial plane for B. (C) Results showing the Maxwell phase accrual along the echo train and during the readout, for a $z_{c}=50\;mm$ off-center axial plane, before (black lines) and after (blue lines) sequence-based compensation. eRF and rRF denote excitation and refocusing RF pulses, respectively. Red dots indicate the k-space center, while orange arrows example the effects of rRF, which alternate the sign of the phase error throughout the echo train. Green dashed boxes indicate examples of increased Maxwell phase by added bipolar gradients, which are played around each readout. After compensation, the center of k-space has zero phase shifts and the phase at the end of each echo spacing has the designed value $\left(\phi \propto \frac{M_{max}}{2}\right)$. (D) Simulation of cross-term Maxwell phase evolutions at a pixel location (y,z)=(50,50) mm in the sagittal plane. The net cross-term phases evolve back to almost zero at the end of each echo spacing when the sequence modification with bipolar-gradient polarity reversal is used (solid line), while there is a large residual cross-term phase difference when bipolar-gradient polarity reversal is not applied (dashed line).

Simulation results in Figure 2A,B illustrate how echo-to-echo phase variations caused by Maxwell fields along the echo train affect the signal pathway of SPRING-RIO TSE without sequence-based compensation. The evolutions of signal intensity loss over the nine echoes, simulated at four different table (z) positions, are shown in Figure 2A. A 150° refocusing RF flip angle was used for the simulation to approximate a slice-selective refocusing RF pulse with a nominal flip angle of 180°. As table position increases, the signal intensity changes along the echo-train in a non-intuitive fashion. For example, signal intensities simulated at 60mm off-center drop rapidly for the first several echoes, while the magnitude of the fifth echo (at ${TE}_{\text{eff}}$) is higher than that of its surrounding echoes, due to the signal distribution among spin echo (SE) and stimulated echo (STE) components. Figure 2B shows the dependence of refocusing flip angles on the magnitude of echoes simulated at 40mm off-center table location. The signal intensity loss curve shows more oscillations as the refocusing flip angle decreases. Note that for a given pixel, echoes from the signal evolution using an ideal 180° refocusing RF pulse (no STE, green line) will have a phase modulation (not shown) which may result in ghosting and shading artifacts, although the magnitudes are the same as those from the standard T_2_-decay curve, which results in no signal intensity loss.

Figure 2C shows the simulation of Maxwell phase pathways from self-squared terms along the echo train and during the acquisition window for SPRING-RIO TSE without (black lines) and with (blue lines) sequence modifications, for an axial plane 50mm off-center. Red dots indicate the k-space center, while orange arrows show the effects of refocusing RF pulses, which alternate the sign of the phase error throughout the echo train. The green dashed boxes indicate examples of increased Maxwell phase by added bipolar gradients. It can be clearly seen that outer rings produce more Maxwell phase accruals than inner rings and thus require less additional Maxwell phase from the added bipolar pairs. After adding the bipolar compensation gradients, the accrued phase for each echo spacing starts at at -ϕ and ends at ϕ, where ϕ is a constant value, and the phase at the k-space center, as well as at the other spin echoes, is zero.

Figure 2D shows the simulation of Maxwell phase evolutions from quadratic cross-terms during the second and central echo spacings for SPRING-RIO TSE without (dashed lines) and with (solid lines) bipolar-gradient polarity reversal, at pixel location (50, 50) mm in the sagittal plane. The quadratic cross-terms from spiral rings have minimal effects on phase variations over echo spacings due to the gradient waveforms with alternating polarities; however, the pair of added bipolar gradients will not only produce the self-squared phase terms as needed, but will also create undesirable cross-terms that may be large enough to induce additional phase error variations among echoes (dashed lines). With bipolar-gradient polarity reversal, the net phase accrual from the bipolars returns to zero at the end of each echo spacing (solid lines).

### 3.2 | Phantom studies

**FIGURE 3:**
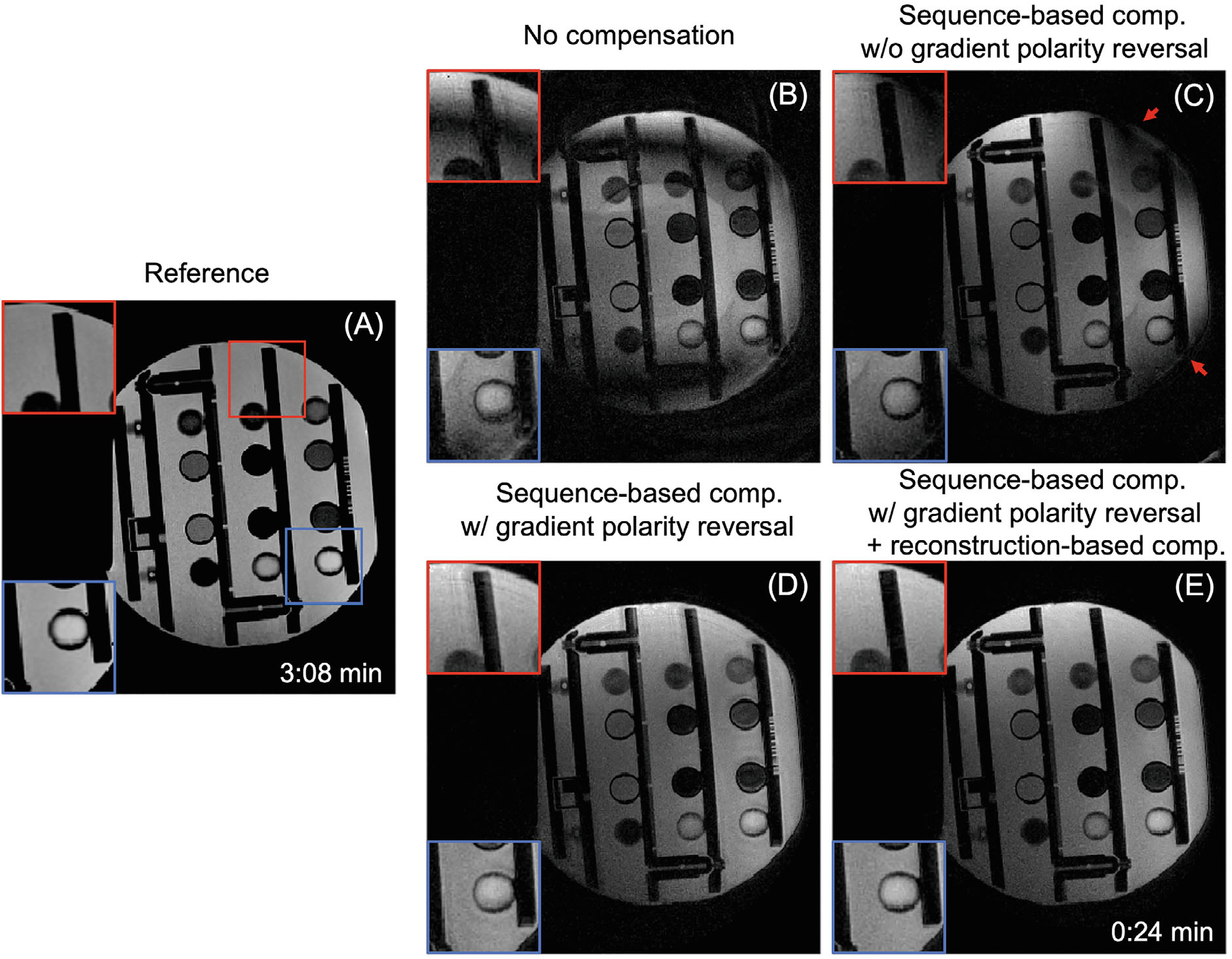
Reconstructed images of a sagittal slice in a resolution phantom demonstrating the performance of the sequence modification with bipolar-gradient polarity reversal on image quality at 0.55T. (A) Image from Cartesian TSE as the reference (scan time: 3:08min). (B–E) Images from SPRING-RIO TSE with different compensation methods (scan time: 0:24 min). (B) Image with no compensation showing severe signal loss and artifacts. (C) Image with sequence-based compensation but without bipolar-gradient polarity reversal, showing improved image quality when compared to B but still displaying obvious artifacts, especially along the diagonals (red arrows). (D) Image with sequence-based compensation including bipolar-gradient polarity reversal, showing much improved image quality with reduced artifacts and signal loss. (E) By also applying image reconstruction corrections for residual phase errors along the readout, the artifacts are further reduced (zoomed regions). Geometric distortion shown in images (B–E) due to gradient nonlinearity could be corrected using standard remapping methods. Image shading shown in images (B–E) could be removed after pre-scan normalization.

Figure 3 shows how sequence modification with bipolar-gradient polarity reversal improves image quality at 0.55T for a sagittal plane at isocenter. Figure 3A is an image from Cartesian TSE as the reference, while Figure 3B is reconstructed from SPRING-RIO TSE with no compensation, showing severe bands of signal loss and obvious image artifacts. Figure 3C is the result with sequence-based compensation, but without bipolar-gradient polarity reversal, showing improved image quality compared to Figure 3B but still presenting noticeable artifacts and signal loss along the diagonals (red arrows), primarily because of cross-term phase errors induced by the added bipolar gradients. Figure 3D is the result with sequence-based compensation, including bipolar-gradient polarity reversal, indicating much better image quality with reduced artifacts and signal loss when compared to Figures 3B, C (see zoomed portions of images indicated by boxes). By applying the image reconstruction-based corrections for residual phase errors during the readout, artifacts are further reduced, as shown in Figure 3E. Some vials may show different image contrast between SPRING-RIO TSE and the reference, mainly due to the different sequence parameters (e.g., ESP and ETL) used in acquisition, though TE and TR are fixed. The images (Figures 3B–E) show slight geometric distortion induced by gradient nonlinearity, which could be corrected using standard remapping methods.^31,32^ Pre-scan normalization could also be utilized to remove the shading in Figure 3B–E.

**FIGURE 4:**
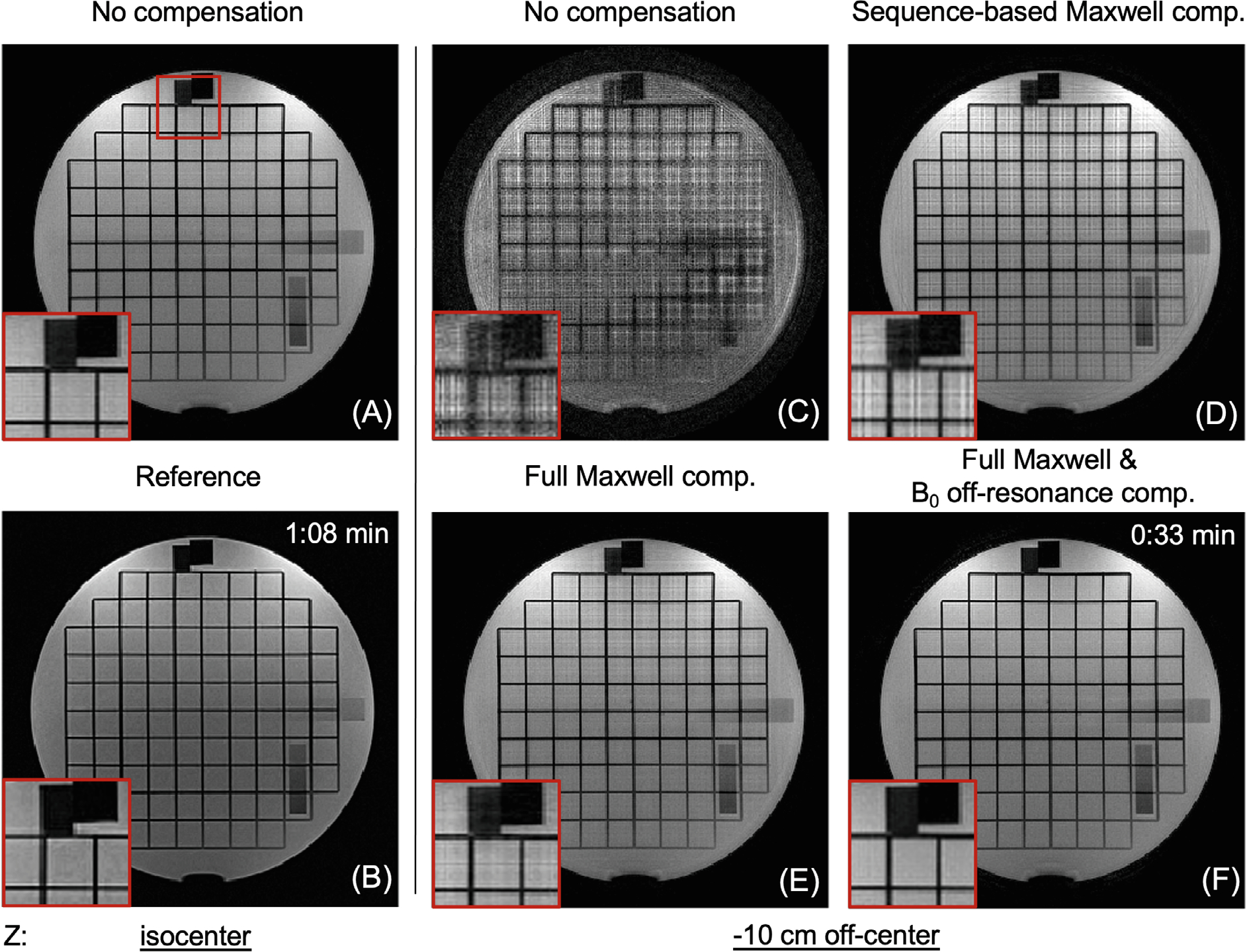
Phantom results from an axial plane scanned at 1.5T showing the performance of concomitant compensation via different sequence modifications and image reconstruction. (A, C–F) Images from SPRING-RIO TSE scanned at two locations and with different compensation methods (scan time: 0:33 min). (B) Image from Cartesian TSE as the reference (scan time: 1:08 min). At isocenter, there are no noticeable artifacts in image (A) from uncompensated SPRING-RIO TSE compared to the reference (B). At −10 cm off-center, the uncompensated image (C) from SPRING-RIO TSE shows substantial signal loss and artifacts. With sequence-based compensation along the echo train (D), no significant signal loss is seen but residual artifacts still exist (zoomed regions). Performing full Maxwell compensation completely removes Maxwell-field-induced image degradation (E). Applying $B_{0}$ inhomogeneity phase correction further improves image quality (F).

Figure 4 shows 1.5T axial images acquired at two table (z) positions using SPRING-RIO TSE with different compensation methods and standard Cartesian TSE. At isocenter (z=0), no obvious image artifacts are seen in Figure 4A from SPRING-RIO TSE with no compensation, compared to the reference in Figure 4B. For =-10cm, however, the result in Figure 4C from SPRING-RIO TSE with no compensation shows severe signal dropouts and blurring. Applying the sequence-based compensation, Figure 4D shows a substantial reduction in image degradation associated with concomitant field effects along the echo train, but residual artifacts still exist. The image quality is further improved by applying reconstruction corrections for residual phase errors along the readout induced by concomitant fields (Figure 4E) and by both concomitant field and $B_{0}$ off-resonance correction (Figure 4F).

**FIGURE 5:**
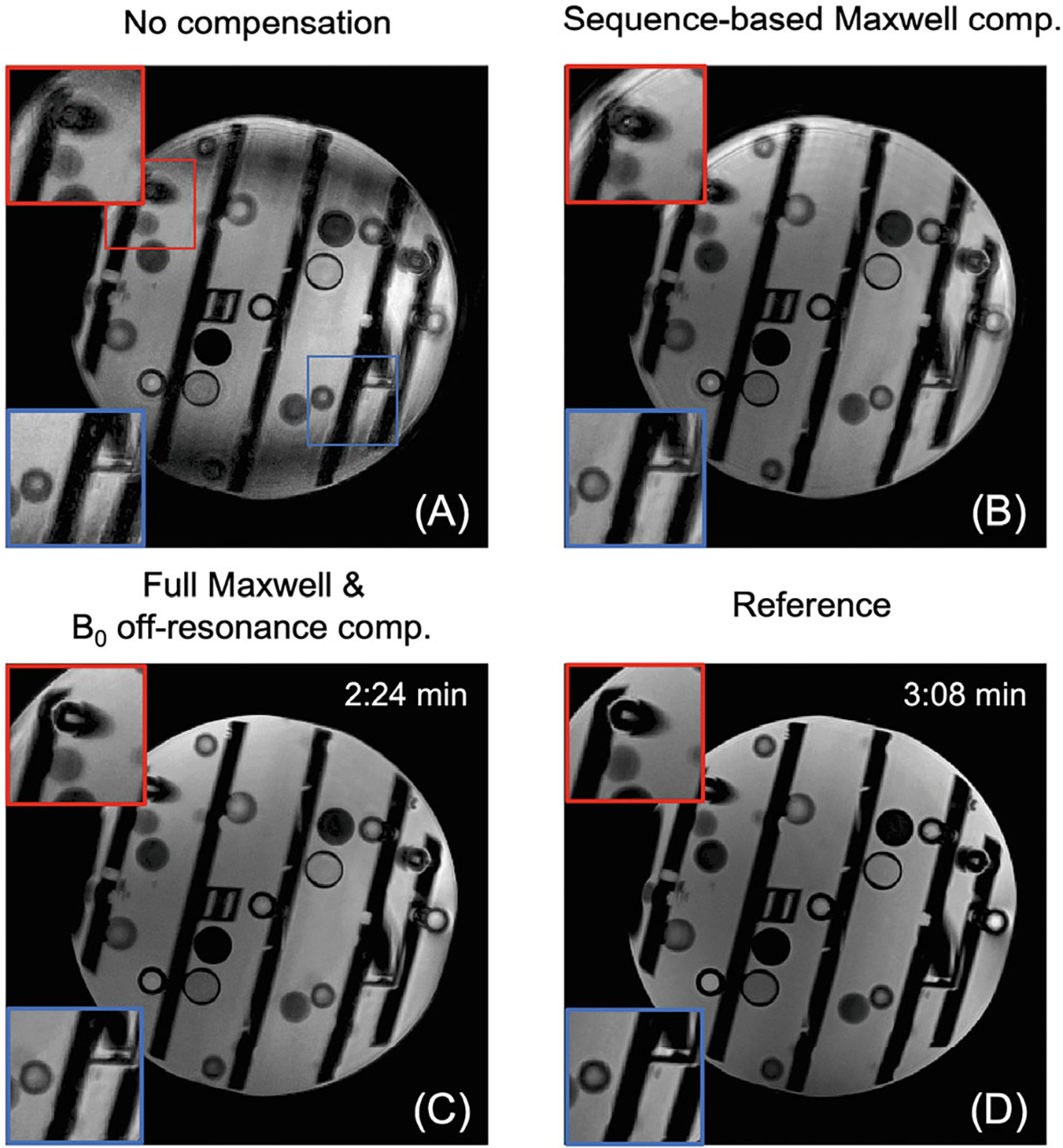
Images of a double-oblique slice (sagittal 30° towards coronal, 20° toward transverse) through a resolution phantom acquired with $G_{max}=21\;mT/m$ at 0.55T. (A-C) Images from SPRING-RIO TSE with different compensation methods (scan time: 2:24 min). (D) Cartesian TSE as the reference (scan time: 3:08 min). Improved image quality, in terms of signal loss, blurring, and artifacts, can be seen in the fully corrected image (C) when compared to uncorrected (A) or partially corrected (B) images (zoomed regions).

Figure 5 shows a representative example of double-oblique phantom images at 0.55T. Improved image quality, in terms of signal loss, blurring, and artifacts, can be seen in the fully corrected image (Figure 5C) when compared to no compensation (Figure 5A) or partially corrected (Figure 5B) images (zoomed regions). Minor residual artifacts and blurring remain in Figure 5C after corrections compared to the reference (Figure 5D), which can also be seen in Figure 3 (Figure 3A vs. Figure 3E), potentially due to quadradic cross-terms or higher order terms from spiral rings during the readout that may need to be further corrected.

### 3.3 | In vivo studies

**FIGURE 6:**
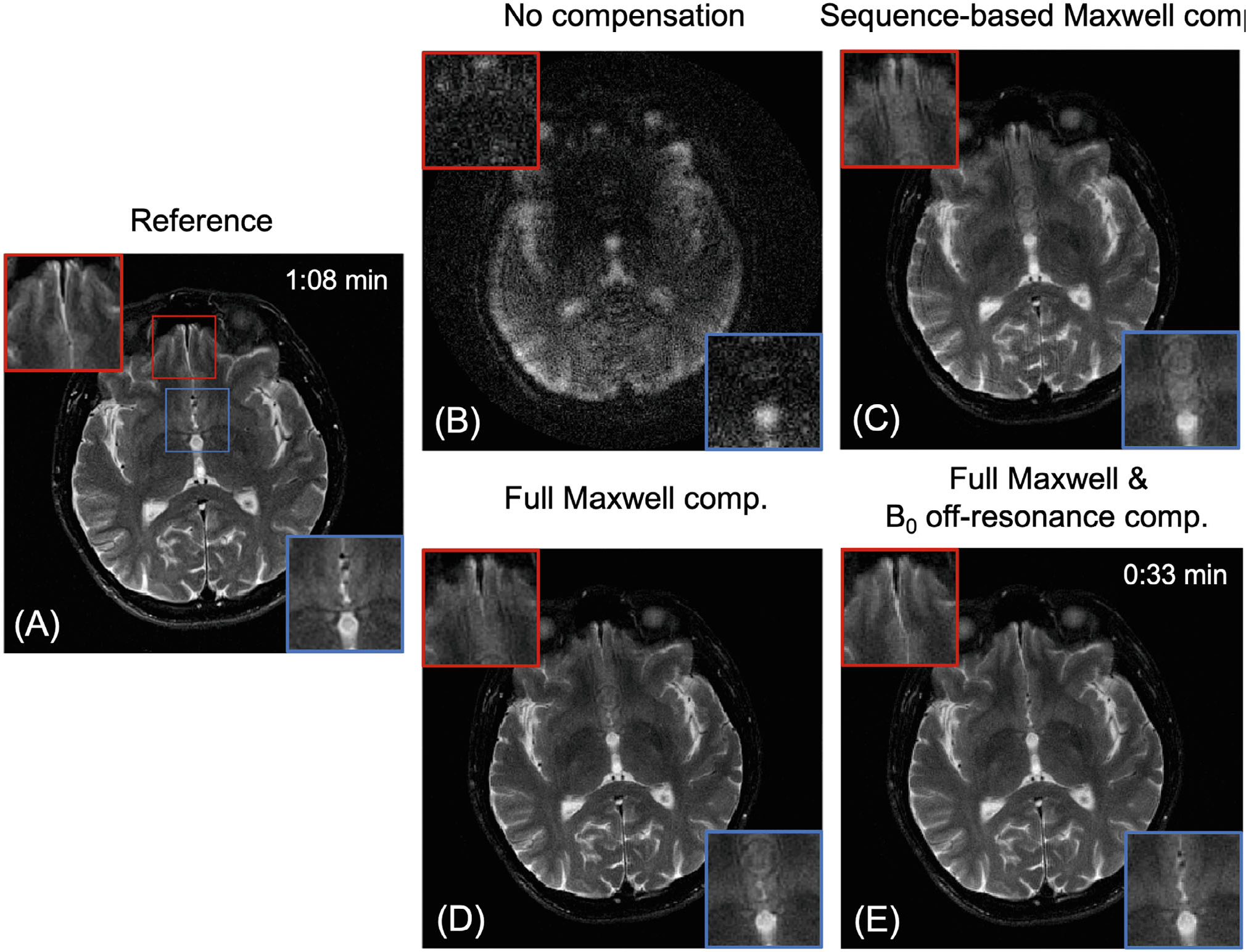
Comparison of 1.5T axial brain images acquired at z=-10.6cm via Cartesian TSE as the reference (A) and SPRING-RIO TSE (B–E). Images are reconstructed with no compensation (B), with sequence-based compensation (C), with full Maxwell field compensation (D) which includes sequence-based compensation along the echo train and reconstruction-based compensation along the trajectory, and with full Maxwell field compensation plus $B_{0}$ off-resonance correction (E). After full corrections, the image E from SPRING-RIO TSE with minimal artifacts presents similar image quality compared to the reference A (zoomed regions) but with less than half the total scan time.

Figure 6 shows images of one 1.5T axial brain slice from a human subject acquired at −10.6 cm off-center. Figure 6A shows the Cartesian TSE image, while Figures 6B–E shows images acquired with the SPRING-RIO TSE sequence with no compensation (Figure 6B), with sequence-based compensation along the echo train (Figure 6C) and reconstructed using full Maxwell field compensation Figure 6D), and full Maxwell field compensation plus $B_{0}$ off-resonance compensation (Figure 6E). Comparing images before and after sequence-based compensation (Figure 6B vs. 6C), improved image quality is consistent with simulation results and phantom studies, showing that strong artifacts and signal dropouts caused by concomitant fields along the echo train can be substantially reduced by the sequence modifications of Figure 1B. Further improvements can be achieved after fully correcting for both concomitant and $B_{0}$ inhomogeneity fields, as seen in Figure 6E compared to Figures 6C,D in the zoomed portions of images indicated by boxes. Another example of an inferior brain slice comparing the performance of reconstruction-based corrections for SPRING-RIO TSE with sequenced-based compensation can be found in Figure S2. After all corrections, SPRING-RIO TSE (0:33 min for 14 slices) has similar image quality compared to the Cartesian reference (1:08 min for 14 slices) but requires less than half of the total scan time.

**FIGURE 7:**
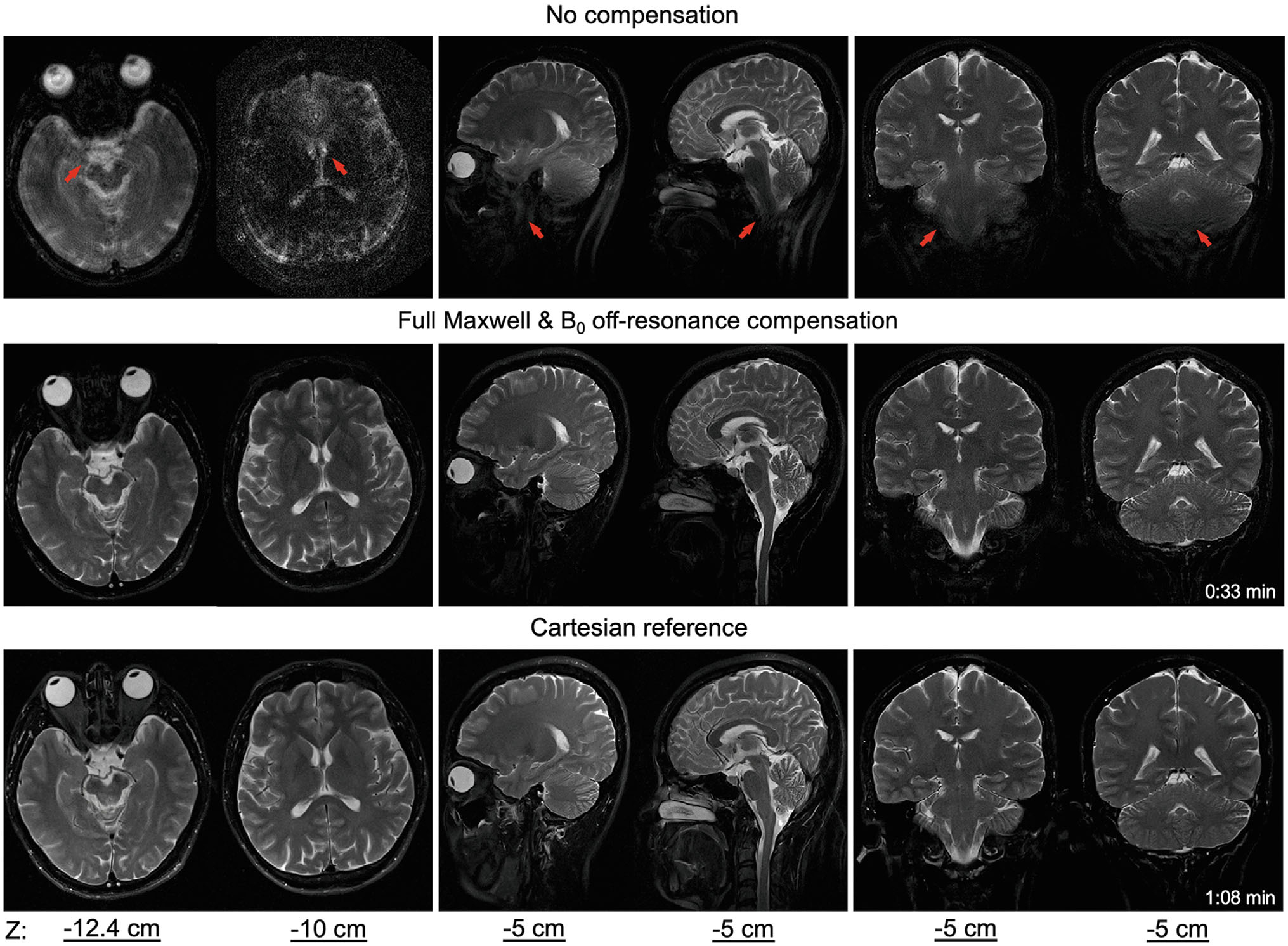
In vivo images acquired at 1.5T using SPRING-RIO TSE with no compensation (top row) or with full Maxwell field compensation plus $B_{0}$ off-resonance compensation (middle row), compared to those from Cartesian TSE (bottom row). The slice positions for axial images (left column) were −12.4 cm and −10 cm, while the table position for sagittal (middle column) and coronal images (right column) was −5 cm. The red arrows indicate structures where SPRING-RIO TSE with no compensation shows strong signal loss, image blurring or artifacts, due to strong time-varying and spatially dependent concomitant gradients.

Figures 7 (1.5T) and 8 (0.55T) display examples of axial, coronal, and sagittal brain images from SPRING-RIO TSE with no compensation (top row) or with full Maxwell plus $B_{0}$ off-resonance compensation (middle row), and from standard Cartesian TSE (bottom row). No obvious artifacts are observed in the SPRING-RIO TSE images after full compensation. SNR measurements in white and gray matter with SPRING-RIO TSE versus the standard Cartesian TSE are shown in Figure 9. Significant increases (15%–20%) of the SNR efficiency (*p* < 0.05) are shown in both white and gray matter for SPRING-RIO TSE compared to those for standard Cartesian TSE at both 0.55T and 1.5T. Examples of the SNR efficiency maps can be found in Figure S3.

## 4 | DISCUSSION

TSE imaging relies heavily on a stable signal pathway along the echo train, and any signal cancellation due to echo-to-echo phase variations from non-negligible, time-varying concomitant fields cannot be corrected by directly applying image-reconstruction-based compensation. Here we demonstrated a spiral annular ring TSE sequence with both sequence- and reconstruction-based correction of concomitant field artifacts at 0.55T and 1.5T. The first and most important step is to eliminate the difference in phase shifts among echoes via sequence modifications to maintain the CPMG condition in SPRING-RIO TSE. Using bipolar waveforms as the quadratic nulling gradients permits the self-squared concomitant field integrals to be further increased, as needed for echoes with small concomitant integrals generated from inner spiral ring waveforms, while still maintaining the original zeroth gradient moment. When imaging in non-axial orientations, additional considerations need to be taken for quadratic cross-terms from non-zero, time-overlapped gradient waveforms. In this work, several strategies were used to mitigate cross-term phase errors. First, the timing of gradient waveforms was adjusted so that there was minimal or no gradient overlapping between slice crushers and readout gradients. Second, unlike spiral-ring readouts, for which the induced cross-term phase can be neglected compared to the self-squared counterparts because of the time-alternation of gradient polarities (see Figure S1), the cross-terms from added bipolar pairs are much closer in amplitude to the self-squared terms, because the same gradient polarity is played during the time of overlap. The performance of artifact suppression using sequence-based compensation with bipolar-gradient polarity reversal for one of four bipolar gradients (Figure 1B) over that without bipolar-gradient polarity reversal (Figure 1A) was demonstrated by the phantom study shown in Figure 3. Although in this study we did not notice a strong influence of small, but non-zero, cross-term phase errors from spiral-ring waveforms, a future study may be needed to evaluate the potential impact of these phase errors on image quality. On the other hand, the added bipolar gradients will create unwanted eddy currents and increase certain sensitivity to flow signals. However, the induced eddy currents will be partially self-canceled with minimal effects on the phase variation over echo spacings because of short, multiple on and off correction gradient transitions. Using a flow-compensated 1–2-1 gradient scheme instead of 1–1 bipolars would show better flow artifacts suppression, although it might further increase the minimum ESP.

**FIGURE 8:**
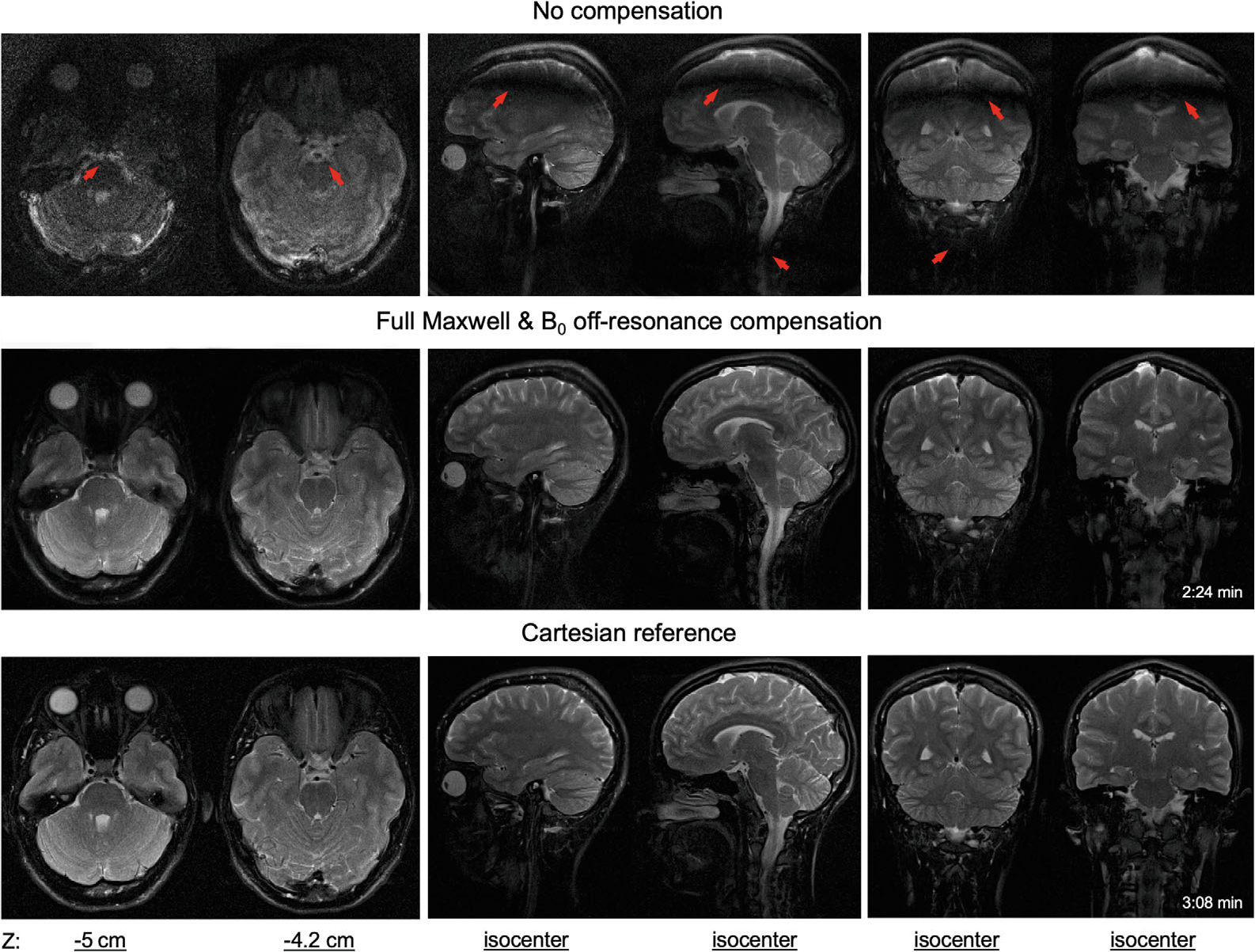
In-vivo images acquired at 0.55T using SPRING-RIO TSE, with no compensation (top row) or with full Maxwell field compensation plus $B_{0}$ off-resonance compensation (middle row), compared to those from Cartesian TSE (bottom row). The slice positions for axial images (left column) were −5 cm and −4.2 cm, while the table position for sagittal (middle column) and coronal images (right column) was isocenter. The red arrows point to structures where there are severe bands of signal loss, image blurring, or artifacts.

Imaging plane shift (or off-center distance) is one of major sources in generating concomitant fields, and for slices that are far away from isocenter, concomitant gradient correction becomes increasingly important. Setting the table position at, or close to, isocenter before scanning is a straightforward way to mitigate concomitant field effects at 1.5T by decreasing the $z^{2}$ component, which dominates the amplitude of the concomitant fields $B_{c}$ in Eq. 1. However, lower-field systems will suffer from image degradation associated with these gradients at positions closer to isocenter than higher-field systems, as the artifacts at 0.55T can be easily seen in Figure 8 where the table position is only 4 cm off-center for an axial plane, and even at isocenter for sagittal and coronal planes. Furthermore, the multi-slice technique used in 2D TSE-based acquisitions may produce undesirable and unexpected artifacts associated with the slice position at 0.55T, which cannot be fixed by a simple table movement to isocenter. Therefore, the proposed compensation method is necessary to mitigate the concomitant gradient effects for a given scan orientation, especially when scanning at low fields.

The maximal amplitude of spiral ring gradients is another concern because the concomitant field phase is proportional to the square of the gradient amplitude. In this work, a moderate maximal gradient amplitude of 21 mT/m was used to constrain the spiral design.^33^ Reducing the maximal gradient amplitude to around 10 mT/m, for example, will decrease the concomitant field offset frequency by a factor of four and thus may alleviate the requirement for comprehensive concomitant field compensation. However, this may increase the total scan time by nearly half per measurement for a given parameter set (FOV, spatial resolution, ETL, etc.). For time-limited applications where imaging speed is an important metric, such as breath-held single-shot T_2_-weighted abdominal^34^ or lung imaging,^35^ decreasing the maximal gradient amplitude for certain reduction of concomitant field effects but with an increased scan time may be impractical; instead, a spiral readout with a higher maximal gradient amplitude (30–40 mT/m) and a higher slew rate (150–180T/m/s) is preferable for fast scanning, and this will inevitably increase the concomitant field offset frequency by several times and must be corrected by the methods proposed in this study.

As observed from phantom and in vivo results, effective image reconstruction-based compensation is achieved to correct the residual phase errors induced by both the concomitant field and $B_{0}$ field inhomogeneity during the spiral-ring readout for each echo, although the retraced in-out design will partially self-correct the off-resonance effects. As discussed in Ref. [24], semiautomatic $B_{0}$ off-resonance correction may offer some compensation for concomitant field effects, since the time parameter t in off-resonance phase term $\phi \left(\Delta \omega \left(r\right),\Delta \omega_{c}\left(r\right),t\right)$ is equivalent to approximating the scaled concomitant field time parameter $t_{c}$ as at, where a is a constant. However, in this work, we found that directly applying semiautomatic $B_{0}$ off-resonance correction to images acquired using SPRING-RIO TSE with sequence-based compensation may still exhibit obvious image artifacts and cannot completely eliminate the residual concomitant field phase error (see Figure S2). The rationale behind this is that the signal phase evolution during the readout caused by concomitant gradients may be very different from that caused by $B_{0}$ inhomogeneity fields in SPRING-RIO TSE. For example, $t_{c}\approx t$ may only fit to outer spiral rings where the gradient amplitude achieves a constant and almost maximum value $g_{m}$, while $t_{c}$ becomes much smaller than the value of t for inner rings with small gradient amplitudes. Thus, the approximation of the scaled time parameter $t_{c}$ as a linear function of t for the same constant parameter a may be unreasonable for spiral rings designed in our sequences, and compensation of time-varying, spatially dependent concomitant fields is necessary before applying semiautomatic $B_{0}$ off-resonance correction. As a result, reconstruction-based compensation was implemented in this work for artifact-free images by simultaneously correcting concomitant gradient field and $B_{0}$ field inhomogeneity.

**FIGURE 9:**
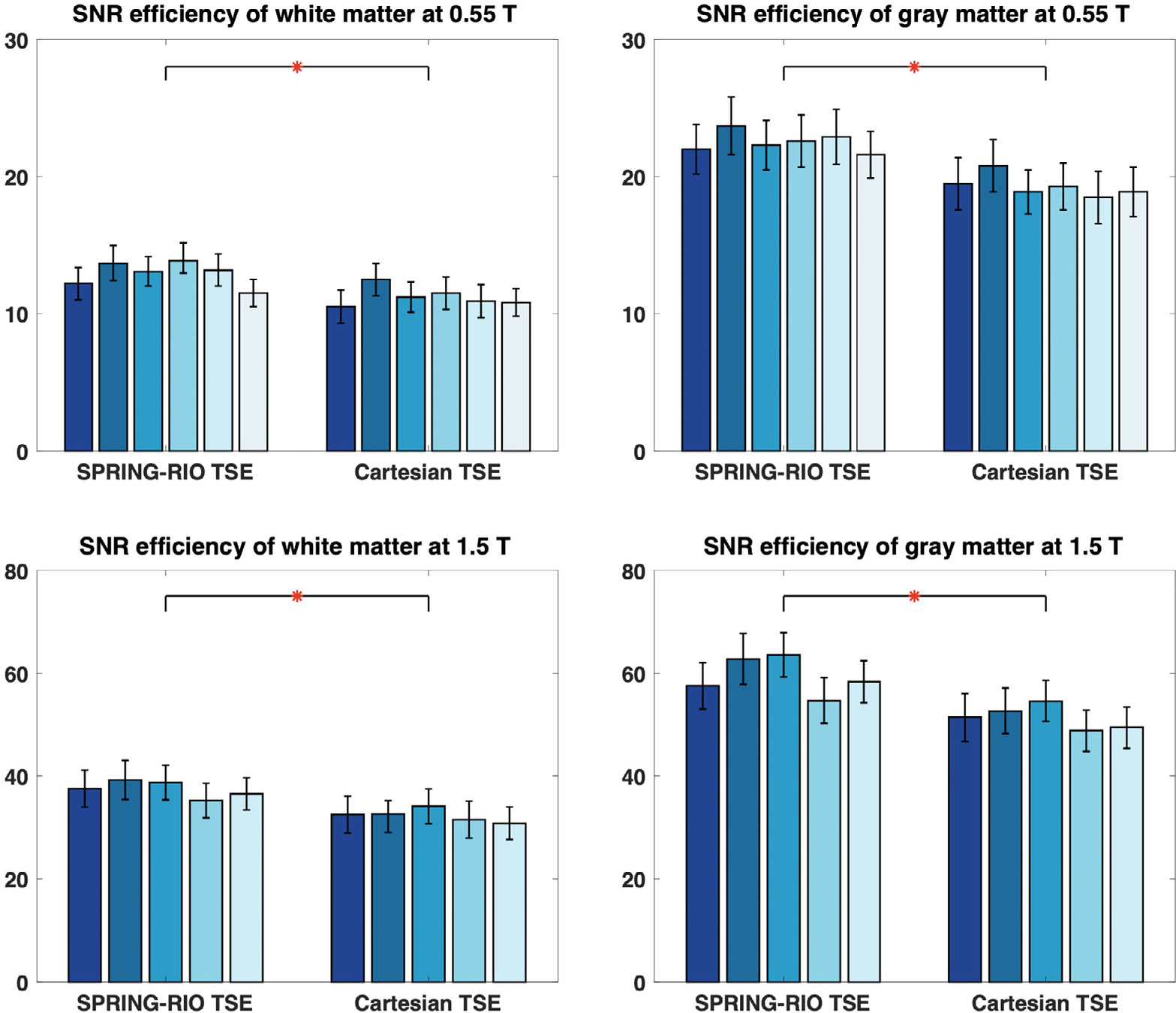
SNR efficiency values of ROIs in white matter (left) and gray matter (right) using SPRING-RIO TSE and standard Cartesian TSE. The different bars for each scenario represent the average values computed for six volunteers at 0.55T (top) and five volunteers at 1.5T (bottom). For each volunteer, nine slices are selected for SNR measurements. The asterisks indicate statistically significant increases of the SNR efficiency for SPRING-RIO TSE over that for Cartesian TSE, in both white matter and gray matter and at both 0.55T and 1.5T (*p* < 0.05).

Compared to imaging at 3T, a lower magnetic field strength (e.g., 0.55T, 1.5T) offers favorable physical properties, such as increased $B_{0}$ field homogeneity and longer $T_{2}^{*}$ decay, which permits SNR-efficient acquisitions such as spiral imaging with a longer readout. Because the SNR efficiency scales with the time spent on sampling but inversely with the total scan time $\left(\propto \sqrt{\frac{T_{\text{sampling}}}{T_{\text{scan}}}}\right)$, a prolonged spiral-ring data acquisition strategy used in this work (e.g., 12 ms at 1.5T, 21 ms at 0.55T) can be leveraged to mitigate the SNR loss at lower fields compared to the standard Cartesian TSE for a fixed total scan time, as demonstrated in Figure 9. Furthermore, for SPRING-RIO TSE protocols shown in Table 1, the RF SAR from the 180° refocusing RF pulses is approximately 53% (e.g., at 0.55T) of that from the corresponding Cartesian TSE, because of a higher k-space coverage per spin echo with a shorter echo train length (e.g., 8 vs. 15) per shot. Increasing the spiral-ring readout duration and/or using a longer ETL is feasible to further improve scan efficiency, though it may induce stronger off-resonance effects and/or an increased RF SAR. However, the sequence-based compensation proposed in this work requires almost 5 ms of additional time added in each echo spacing; future studies are warranted to explore optimization of sequence parameters to reduce the sacrifice in SNR/scan efficiency.

## 5 | CONCLUSIONS

We demonstrated a 2D spiral-ring T_2_-weighted TSE pulse sequence that incorporates sequence modifications and image reconstruction methods to mitigate image degradation associated with concomitant gradient effects at 0.55T and 1.5T. This approach presents a general compensation method that can be extended to compensate concomitant field induced effects for TSE imaging with other asymmetric non-Cartesian trajectories for low-field MRI and is applicable to all field strengths.

## Supplementary Material

Supplementary Figures **Figure S1.** Simulation of phase evolutions induced by Maxwell fields for one specific pixel in the sagittal plane, for which the parameters include $B_{0}=0.55\;T,\;Gmax\;=21\;mT/m,\;ETL=9,\;spiral-ring\;duration=18\;ms$, and pixel location (y,z)=(50,50)mm. The self-squared term produced by the first spiral ring with the largest gradient amplitude is almost five times larger than that produced by the central ring (blue solid line vs. orange solid line). The self-squared term from either the outer ring or inner ring is substantially larger than its corresponding quadratic cross-term (blue solid line vs. blue dashed line, orange solid line vs. orange dashed line, respectively).
**Figure S2.** Reconstructed images of an axial brain slice from Cartesian TSE (A) and SPRING-RIO TSE (B–E) and at 1.5T. (B) Image acquired with sequence-based compensation but without any reconstruction compensation. (C) Image acquired with sequence-based compensation and reconstructed with semiautomatic $B_{0}$ off-resonance compensation only during the readout. (D) Image acquired with sequence-based compensation and reconstructed with Maxwell field compensation only during the readout. (E) Image acquired with sequence-based compensation and reconstructed with simultaneous Maxwell field and $B_{0}$ off-resonance compensation during the readout. Note that artifacts still exist in image C and D, due to the residual off-resonance effects.
**Figure S3.** Example comparisons of the SNR efficiency maps of SPRING-RIO TSE and the Cartesian reference at 0.55 T. The SNR efficiency values of ROIs (1–3 for WM, 4–6 for GM) are shown below.

## Figures and Tables

**TABLE 1:** Sequence parameters for SPRING-RIO TSE and Cartesian TSE at 0.55T and 1.5T

	Sequences	FOV (mm^2^)	TR (ms)	TEeff (ms)	Readout (ms)	Refocusing RF	ESP (ms)	ETL	No. of shots	No. of NSA	Scan time (min)	Spatial res (mm^3^)
0.55T	SPRING-RIO TSE	230 × 230	3000	127	21	180°	31.7	8	8	6	2:24	0.70 × 0.70 × 4
	Cartesian TSE	230 × 230	3000	126	8	180°	12.6	16	20	3	3:08	0.72 × 0.72 × 4
1.5T	SPRING-RIO TSE	230 × 230	3000	114	12	180°	22.8	9	11	1	0:33	0.71 × 0.71 × 4
	Cartesian TSE	230 × 230	3000	117	5	180°	10.7	16	20	1	1:08	0.72 × 0.72 × 4

## APPENDIX A

The derivation of Eq. 7 is described below:

1. After sequence-based corrections, the final concomitant integrals at the beginning and at the end of each echo spacing are constant values of $-M_{max}/2$ and $M_{max}/2$, respectively.
2. Due to the added bipolars, the increased Maxwell integral produced by the added bipolars for the current $j^{\text{th}}$ echo spacing is equal to $\left(M_{max}-M_{j}\right)$, with the Maxwell integrals produced by the bipolars both before and after the readout equal to $\left(M_{max}-M_{j}\right)/2$.
3. The Maxwell integral at the beginning of the readout can be expressed as
  $$-\frac{M_{max}}{2}+\frac{M_{max}-M_{j}}{2}=-\frac{M_{j}}{2}$$
4. The Maxwell integral $M_{j}(t)$ during the readout can then be expressed as
  $$M_{j}(t)=-\frac{M_{j}}{2}+\int_{0}^{t}\;g_{j}^{2}\left(t^{\prime }\right)dt^{\prime }$$
5. The final time parameter $t_{c_{j}}(t)$ can then be calculated by dividing $M_{j}(t)$ by $g_{m}^{2}$, which becomes:
  $$t_{c_{j}}(t)=\frac{1}{g_{m}^{2}}\left(\int_{0}^{t}\;\;g_{j}^{2}\left(t^{\prime }\right)dt^{\prime }-\frac{M_{j}}{2}\right)$$
