# Supplementary material for: Concomitant magnetic-field compensation for 2D spiral-ring turbo spin-echo imaging at 0.55T and 1.5T
Source: Magn Reson Med. Author manuscript; Available in PMC 2024 Aug 1. (PMC10578525; doi:10.1002/mrm.29663)
Supplement: Supplementary Figures — Figure S1. Simulation of phase evolutions induced by Maxwell fields for one specific pixel in the sagittal plane, for which the parameters include B0=0.55T,Gmax=21mT/m,ETL=9,spiral-ringduration=18ms, and pixel location (y,z)=(50,50)mm. The self-squared term produced by the first spiral ring with the largest gradient amplitude is almost five times larger than that produced by the central ring (blue solid line vs. orange solid line). The self-squared term from either the outer ring or inner ring is substantially larger than its corresponding quadratic cross-term (blue solid line vs. blue dashed line, orange solid line vs. orange dashed line, respectively). Figure S2. Reconstructed images of an axial brain slice from Cartesian TSE (A) and SPRING-RIO TSE (B–E) and at 1.5T. (B) Image acquired with sequence-based compensation but without any reconstruction compensation. (C) Image acquired with sequence-based compensation and reconstructed with semiautomatic B0 off-resonance compensation only during the readout. (D) Image acquired with sequence-based compensation and reconstructed with Maxwell field compensation only during the readout. (E) Image acquired with sequence-based compensation and reconstructed with simultaneous Maxwell field and B0 off-resonance compensation during the readout. Note that artifacts still exist in image C and D, due to the residual off-resonance effects. Figure S3. Example comparisons of the SNR efficiency maps of SPRING-RIO TSE and the Cartesian reference at 0.55 T. The SNR efficiency values of ROIs (1–3 for WM, 4–6 for GM) are shown below. [file NIHMS1926329-supplement-Supplementary_Figures.pdf]

Supporting Information

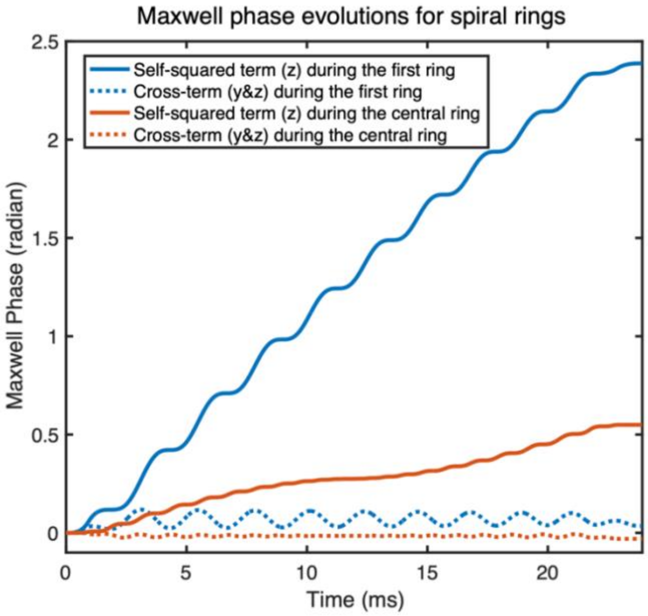

Supporting Information Figure S1. Simulation of phase evolutions induced by Maxwell fields for one specific pixel in the sagittal plane, for which the parameters include B_0 = 0.55 T, $Gmax$ = 21 mT/m, ETL = 9, spiral-ring duration = 18 ms, and pixel location (y, z) = (50, 50) mm. The self-squared term produced by the first spiral ring with the largest gradient amplitude is almost five times larger than that produced by the central ring (blue solid line vs. orange solid line). The self-squared term from either the outer ring or inner ring is substantially larger than its corresponding quadratic cross-term (blue solid line vs. blue dashed line, orange solid line vs. orange dashed line, respectively).

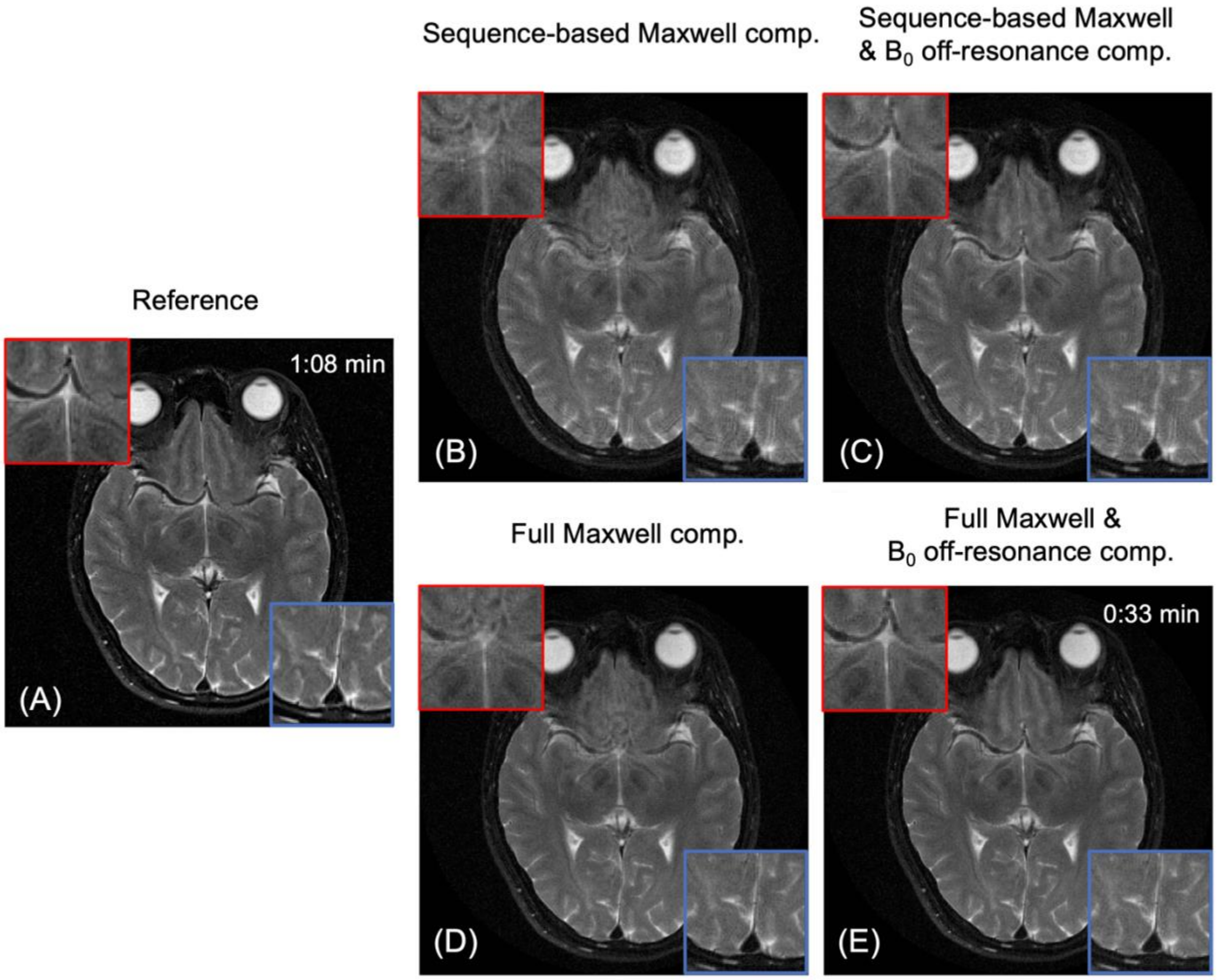

Supporting Information Figure S2. Reconstructed images of an axial brain slice from Cartesian TSE (A) and SPRING-RIO TSE (B-E) and at 1.5 T. B: Image acquired with sequence-based compensation but without any reconstruction compensation. C: Image acquired with sequence-based compensation and reconstructed with semiautomatic B_0 off-resonance compensation only during the readout. D: Image acquired with sequence-based compensation and reconstructed with Maxwell field compensation only during the readout. E: Image acquired with sequence-based compensation and reconstructed with simultaneous Maxwell field and B_0 off-resonance compensation during the readout. Note that artifacts still exist in image C and D, due to the residual off-resonance effects.

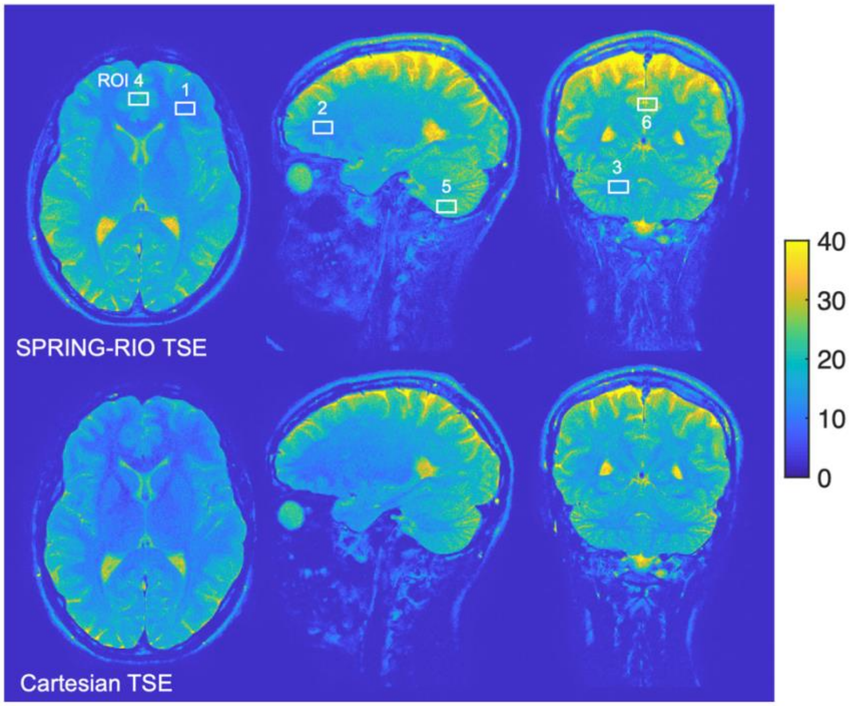

Averaged SNR	White matter			Gray matter		
ROIs	1	2	3	4	5	6
SPRING-RIO TSE	9.1	11.5	13.0	20.9	22.1	25.3
Cartesian TSE	7.6	9.7	10.9	17.5	18.9	20.6

Supporting Information Figure S3. Example comparisons of the SNR efficiency maps of SPRING-RIO TSE and the Cartesian reference at 0.55 T. The SNR efficiency values of ROIs (1-3 for WM, 4-6 for GM) are shown below.
